# Biomarker of Aflatoxin Ingestion: ^1^H NMR-Based Plasma Metabolomics of Dairy Cows Fed Aflatoxin B_1_ with or without Sequestering Agents

**DOI:** 10.3390/toxins10120545

**Published:** 2018-12-18

**Authors:** Ibukun Ogunade, Yun Jiang, James Adeyemi, Andre Oliveira, Diwakar Vyas, Adegbola Adesogan

**Affiliations:** 1 College of Agriculture, Communities, and the Environment, Kentucky State University, Frankfort, KY 40601, USA; james.adeyemi@kysu.edu; 2 Department of Animal Sciences, University of Florida, Gainesville, FL 32611, USA; xtiemira@gmail.com (Y.J.); Diw9268@gmail.com (D.V.); adesogan85@gmail.com (A.A.); 3 Institute of Agriculture and Environmental Sciences, Federal University of Mato Grosso, Sinop, MT 78557-267, Brazil; andresoli@uol.com.br

**Keywords:** aflatoxin, dairy cow, metabolomics, plasma

## Abstract

The study applied ^1^H NMR-based plasma metabolomics to identify candidate biomarkers of aflatoxin B1 (AFB_1_) ingestion in dairy cows fed no sequestering agents and evaluate the effect of supplementing clay and/or a *Saccharomyces cerevisiae* fermentation product (SCFP) on such biomarkers. Eight lactating cows were randomly assigned to 1 of 4 treatments in a balanced 4 × 4 Latin square design with 2 squares. Treatments were: control, toxin (T; 1725 µg AFB_1_/head/day), T with clay (CL; 200 g/head/day), and CL with SCFP (CL + SCFP; 35 g of SCFP/head/day). Cows in T, CL, and CL + SCFP were dosed with AFB_1_ from d 26 to 30. The sequestering agents were top-dressed from d 1 to 33. On d 30 of each period, 15 mL of blood was taken from the coccygeal vessels and plasma samples were prepared by centrifugation. Compared to the control, T decreased plasma concentrations of alanine, acetic acid, leucine, arginine and valine. In contrast, T increased plasma ethanol concentration 3.56-fold compared to control. Treatment with CL tended to reduce sarcosine concentration, whereas treatment with CL + SCFP increased concentrations of mannose and 12 amino acids. Based on size of the area under the curve (AUC) of receiver operating characteristic and fold change (FC) analyses, ethanol was the most significantly altered metabolite in T (AUC = 0.88; FC = 3.56); hence, it was chosen as the candidate biomarker of aflatoxin ingestion in dairy cows fed no sequestering agent.

## 1. Introduction

Aflatoxins are a type of mycotoxins produced by strains of *Aspergillus flavus*, *Aspergillus parasiticus*, and *Aspergillus nominus* on growing forages or stored feeds [1]. Aflatoxin B_1_ (AFB_1_) is the most widely studied and potent carcinogen among all aflatoxins [2]. Up to 6% of its metabolite can be transferred into milk as aflatoxin M_1_ [3]. Aflatoxin ingestion by cows and the resultant aflatoxicosis cause effects such as reduced cow health and performance, impaired liver function, and increased susceptibility to diseases [4]. Several approaches, notably the use of clay-based sequestering agents and *Saccharomyces cerevisiae* fermentation product (SCFP), have been documented to reduce the transfer of aflatoxin into the milk of dairy cows [5,6]. Our recent study revealed that supplementation of diet with both SCFP and clay products was more effective than clay alone at maintaining milk production during aflatoxin challenge [7]. Despite the efficacy of these sequestering agents at reducing the milk aflatoxin concentration, the plasma metabolome of aflatoxin-challenged dairy cows has not been described. Such information is urgently needed to identify biomarkers of aflatoxin ingestion that may allow early detection and mitigation of aflatoxicosis in livestock due to the variable clinical signs of the syndrome that resemble those of other diseases [8]. Currently, diagnosis of aflatoxin ingestion is based on feed mycotoxin analysis, which may be inaccurate considering difficulty in obtaining representative feed sample because of uneven distribution of toxin within the feed [9]. Therefore, studies aiming at identifying biomarkers of aflatoxicosis in ruminants are urgently needed as a similar approach has been successfully applied to determine aflatoxin exposure in humans [10].

Metabolomics is the study of level of small endogenous molecules or metabolites [11]. High-resolution proton nuclear magnetic resonance spectroscopy (^1^H NMR)-based metabolomics can identify and quantify many metabolites, such as amino acids, sugars, alcohols, organic acids, amines, tricarboxylic acid cycle intermediates, and short chain fatty acids in biofluids [12]. Though ^1^H NMR has lower sensitivity than mass spectrometry-based techniques, it provides the most reliable quantitative information of metabolites [13]. In addition, the high analytical reproducibility is a significant advantage of ^1^H NMR over mass spectrometry-based methods, such as gas chromatography mass spectrometry (GC-MS) and liquid chromatography mass spectrometry (LC-MS) [14,15]. Sun et al. [16] demonstrated the utility of ^1^H NMR-based plasma metabolomics for the detection of ketosis in dairy cows. Therefore, the objectives of this study are to use ^1^H NMR-based plasma metabolomics (1) to identify potential biomarker(s) of aflatoxin ingestion in dairy cows fed no sequestering agents and (2) to assess the effects of clay and/or SCFP-based sequestering agents on the plasma metabolome of dairy cows challenged with AFB_1_.

## 2. Results and Discussion

Detailed results on treatment effects on dairy cow performance and milk aflatoxin concentration were reported in our companion paper [7]. Briefly, CL and CL + SCFP reduced (*p* < 0.01) transfer of dietary AFB_1_ into milk. Dietary supplementation with CL + SCFP tended (*p* < 0.10) to increase the milk yield of the cows compared with T.

**Figure 1:**
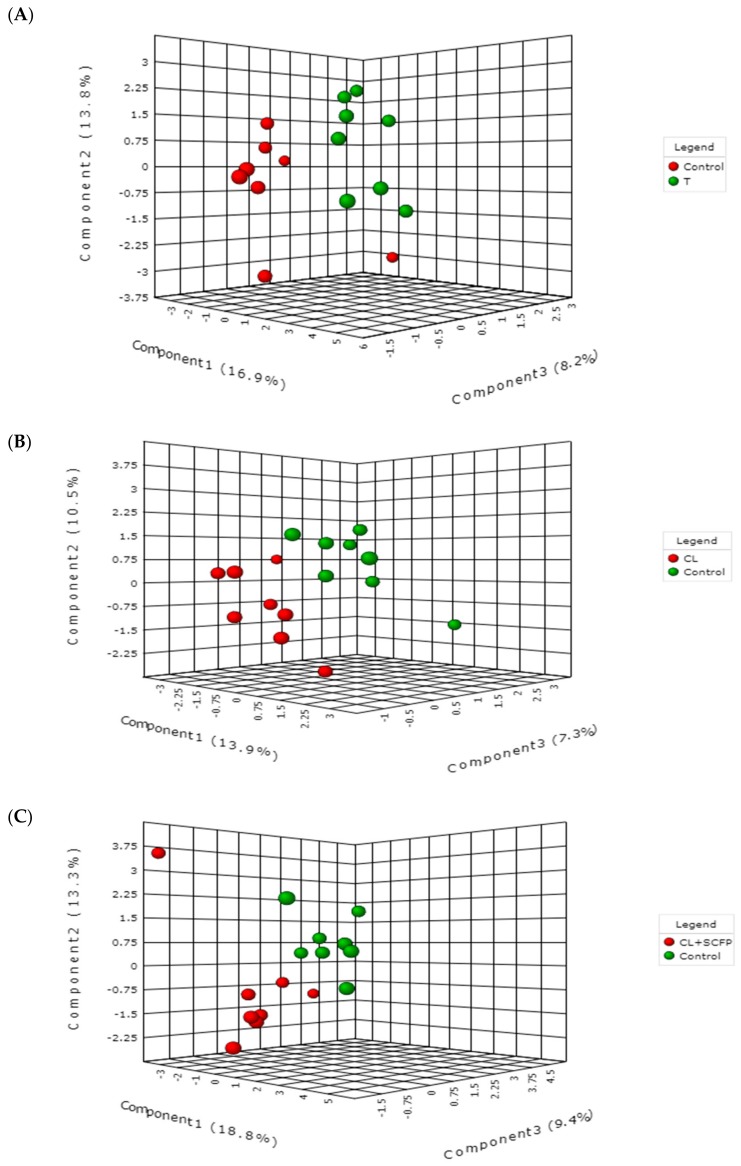
Partial least squares-discriminant analysis showing 2 clusters for Control and Toxin (T) groups (**A**), Control and Clay (CL) groups (**B**), and Control and CL + *Saccharomyces cerevisiae* fermentation product (SCFP) groups (**C**). Percentages of variance explained by each component are presented in parentheses on the axes.

A total number of 45 metabolites were identified and quantified (Table S1). The scores plot of the partial least squares discriminant analysis (PLS-DA; Figure 1) revealed good separations between the control group and each of T, CL, and CL + SCFP groups, indicating that dietary treatment altered the plasma metabolome relative to the control. The corresponding loading plots are presented in Figure S1. The respective *p* values for 2000 permutation were 0.09, 0.07, and 0.02, indicating the validity of the PLS-DA models.

Table 1 provides the results of the metabolites that were affected by dietary treatment. Compared to the control, T diet decreased (*p* < 0.05) plasma concentrations of acetic acid, alanine and leucine, tended to decrease (*p* < 0.10) that of arginine and valine, and increased (*p* = 0.01) plasma ethanol concentration.

It is well documented that AFB_1_ impairs animal and human health by interfering with substrates and enzymes needed for initiation and translation in protein synthesis and/or form adducts with DNA, RNA and proteins [17,18]. Leucine and valine have been shown to be major stimulators of protein synthesis through the mechanistic target of rapamycin (mTOR) signaling cascade, a major regulator of protein synthesis at the point of translation initiation [19]. A master role of mTOR in the regulation of protein synthesis in all tissues of mammals, including the mammary gland, has been well defined [20]. This probably explains the reason for the suppressed immune system of animals exposed to aflatoxins [21] because proteins make up part of antibodies, interferon and complement proteins that support immune system cells. The fact that milk protein concentration of the cows was not affected by dietary aflatoxin, as shown in our companion paper [7], is probably because the glucose level, which drives milk protein synthesis [22], and lysine, methionine, and histidine, largely considered the limiting amino acids for the synthesis of protein in the mammary glands [23], were not affected by AFB_1_.

The results of receiver operating characteristic (ROC) analysis revealed that plasma acetic acid, arginine, ethanol, alanine, methylhistidine, and proline were candidate biomarkers of aflatoxin ingestion in dairy cows fed no sequestering agents according to AUC > 0.80 (Table 2). Based on the results of AUC > 0.80 and FC analyses, ethanol was found to be the most significantly altered metabolite (AUC = 0.88; FC = 3.56) in the dataset and was chosen as the most important candidate biomarker of aflatoxin ingestion in this study.

Dairy cows without metabolic stress can metabolize alcohols present in feeds, particularly silage-based feeds, because the liver has a high affinity for alcohol and will primarily metabolize alcohols via alcohol and aldehyde dehydrogenases to acetate [24]. Exposure to aflatoxins in diets of ruminants has been reported to cause impaired liver function [25]. Though effects of AFB_1_ on liver alcohol dehydrogenase have not been reported in dairy cows, AFB_1_ has been shown to cause downregulation of genes from alcohol and aldehyde dehydrogenase groups in domesticated and wild turkey [26]. In humans, dysregulation of alcohol dehydrogenase activity has been shown to be associated with a risk of liver dysfunction and cancer [27]. Increased alcohol concentration and decreased acetate concentration in plasma of cows fed AFB_1_ probably suggest reduced activity of alcohol dehydrogenase in the liver, which indicates an impaired liver function. More studies are needed to confirm the effects of AFB_1_ on enzymes (alcohol and aldehyde dehydrogenases) responsible for alcohol metabolism in the liver of dairy cows. It is expected that biomarkers of aflatoxin ingestion in livestock are likely to differ, depending on the cattle feeds. Future studies with larger animal groups, as well as integration of NMR and mass-spectrometry techniques that can offer a powerful methodology for metabolomics studies, are needed to identify biomarkers of aflatoxin ingestion in livestock across different feeding regimens.

Dietary treatment with CL did not affect any of the plasma metabolites, except plasma sarcosine, which was reduced (*p* = 0.07) compared to control diet. This suggests that clay supplementation was effective at preventing the inhibitory effects of AFB_1_ on protein synthesis and liver function. The role of sarcosine, an intermediate in the conversion of choline to glycine [28], on the health status of dairy cows has not been described; however, it was reported to promote neuroprotection in rats [29]. Reduced level of serum sarcosine was observed in dairy cows with foot rot [30] and in dairy cows with short productive life due to various health and fertility problems such as metabolic disorders, inflammatory production diseases, infertility, and low conception rates [31]. However, the extent of sarcosine reduction with CL in this study is small (1.83 vs. 1.50 µM) and may not be of biological relevance.

Dietary treatment with CL + SCFP increased (*p* < 0.05) or tended to increase (*p* < 0.10) the plasma concentrations of mannose, essential amino acids, such as threonine, valine, lysine, arginine, leucine, isoleucine, and phenylalanine, and non-essential amino acids, such as l-glutamic acid, l-alanine, proline, serine, and aspartic acid. The increased plasma amino acid and mannose concentration probably reflects the effect of SCFP alone because similar results were not observed with CL treatment. These results explain the results of our companion paper [7], which showed that CL + SCFP was more effective than CL alone at increasing the milk and milk protein production of cows fed the toxin. In support of this study, Leicester et al. [32] observed increased plasma concentration of amino acids, such as threonine, valine, proline, and serine of dairy cows fed a yeast additive. The concentration of amino acid in the plasma is a representation of intestinally-absorbed amino acid [33]. Several studies have reported increased duodenal flow of amino acids in cows fed yeast additive [32,34]. The increase in duodenal amino acid is attributable to increased flow of microbial protein from the rumen and improved nutrient absorption efficiency because SCFP has been shown to increase rumen bacterial number [35] and feed efficiency [36], and improve gut health in the small intestine [32].

In the cell wall of *S. cerevisiae*, mannan oligosaccharides (MOS) are present in complex molecules that are linked to a protein moiety [37]. MOS contains d-mannose, which reduces the risk of pathogen colonization in the gut by binding to pathogens, such as *E. coli* and *Salmonella* spp., via type-1-fimbriae [38]. d-mannose is non-fermentable by some groups of bacteria in the rumen and is resistant to enzymatic activity in the gut [39]. This explains the increased plasma mannose concentration in cows fed CL + SCFP. Mannose plays an important role in the mannosylation of proteins such as mannose-binding lectin, which plays a critical role in innate immune system function [40]. Mannose-binding protein is one of the soluble pattern recognition molecules that activate innate immune cells [41]. This supports the immunostimulatory effects of SCFP against toxins and stress reported by several studies [42,43]; however, no effect on immune status was observed in our companion study, probably because the level of AFB_1_ (1725 µg/cow) and duration of the challenge (5 d) were not adequate to suppress the immune status of the cow [7].

In conclusion, dosing AFB_1_ altered the plasma metabolomic profile of lactating Holstein dairy cows by decreasing plasma concentrations of alanine, leucine, arginine, acetic acid and increasing concentration of ethanol. Plasma ethanol concentration was found to be 3.56-fold higher in aflatoxin-challenged cows compared to the control, and was chosen to be a candidate biomarker of aflatoxin ingestion in dairy cows under the experimental conditions of the present study. Sodium bentonite clay prevented the adverse effects of AFB_1_ on the metabolic status of the cows and CL + SCFP improved the metabolic status of the aflatoxin-challenged cows compared to the control cows.

## 3. Materials and Methods

The animal trial protocol was reviewed and approved by the Institutional Animal Care and Use Committee of University of Florida (IACUC Protocol #: 201509099, Date of approval: 13 January 2016).

### 3.1. Animals, Housing, and Feeding

The experiment was part of a larger study that evaluated the effect of supplementing clay with or without a SCFP on the performance and health of dairy cows challenged with AFB_1_. Detailed information about cows and feeding has been reported in our companion paper [7]. Briefly, eight lactating multiparous Holstein cows in early lactation (64 ± 11 days in milk) were stratified by parity and milk production and randomly assigned to 1 of 4 treatment sequences arranged in a balanced 4 × 4 Latin square design with 2 replicate squares, four 33-d periods, and a 5-d washout interval between periods. Dietary treatments were (1) control (basal diet without additives), (2) toxin (T; oral dose of 1725 µg of AFB1/head per day), (3) toxin with bentonite clay (CL; 200 g/head per day; Astra-Ben-20, Prince Agri Products Inc., Quincy, IL, USA), and (4) CL plus SCFP (CL + SCFP; 35 g of SCFP/head per day; Diamond V, Cedar Rapids, IA, USA). The basal diet was fed as a total mixed ration (TMR) containing, on a dry matter (DM) basis, 36.1% corn silage, 8.3% alfalfa hay and 55.6% concentrate mix. The TMR was formulated to meet or exceed nutrient requirements of lactating dairy cows producing at least 30 kg/d of milk [44] with CPM-Dairy version 3.0.10 software (www.cpmdairy.net).

Cows in treatments T, CL, and CL + SCFP were orally dosed with 1725 µg of AFB_1_/head per day from day 26 to 30 before the morning feeding to give a dietary concentration of 75 µg/kg of DM based on estimated daily DMI of 23 kg/d. The sequestering agents were top-dressed on the respective TMR from day 1 to 33 of each period. Aflatoxin B1, obtained from the University of Missouri Diagnostic Laboratory (Columbia, MO, USA), was mixed with 10 g of ground corn and 4 mL of molasses and then weighed into gelatin capsules before orally dosing each cow daily. Control cows were provided with similar quantities of ground corn and molasses without AFB_1_. A 5-d washout period was imposed after each withdrawal period to minimize carryover of treatment effects between periods.

Subsamples of the basal diet and ground corn used in this study were initially analyzed for AFB_1_, ochratoxin, zearalenone, and deoxynivalenol using HPLC at the University of Missouri Veterinary Diagnostic Laboratory. These mycotoxins were below lower detection limits (5 µg/kg for aflatoxins; 0.5 mg/kg for ochratoxin, zearalenone, and deoxynivalenol).

### 3.2. Blood Sample Collection

On day 30 of each experimental period, 15 mL of blood was taken from the coccygeal vessels into vacutainer tubes containing sodium heparin anticoagulant. Plasma samples were immediately prepared by centrifugation at 2500× *g* for 20 min at 4 °C, and stored at −20 °C until NMR analysis was done.

### 3.3. Sample Preparation and NMR Spectroscopy

A deproteinization step, involving ultra-filtration, previously described by Psychogios et al. [45], was used to remove plasma lipoproteins and proteins with large molecular weight, which might affect the identification of the small molecular weight metabolites. Prior to filtration, 3 kDa cut-off centrifugal filter units (Amicon Microcon YM-3, Sigma-Aldrich, St. Louis, MO, USA) were rinsed five times each with 0.5 mL of H_2_O and centrifuged (10,000 rpm for 10 min) to remove residual glycerol bound to the filter membranes. Aliquots of each plasma sample were then transferred into the centrifuge filter devices and spun (10,000 rpm for 20 min) to remove macromolecules (primarily protein and lipoproteins) from the sample. The subsequent filtrates were collected and the volumes were recorded. Subsequently, 160 µL of the sample was mixed with 40 µL of a standard buffer solution (54% D_2_O:46% 250 mM KH_2_PO_4_, pH 7.0). The sample (200 µL) was then transferred to a 3 mm SampleJet NMR tube for subsequent spectral analysis. All ^1^H-NMR spectra were collected on a 700 MHz Avance III (Bruker, Billerica, MA, USA) spectrometer equipped with a 5 mm HCN Z-gradient pulsed-field gradient cryoprobe. The ^1^H-NMR spectra were acquired at 25 °C using the first transient of the nuclear overhauser effect spectroscopy (NOESY) pre-saturation pulse sequence, chosen for its high degree of quantitative accuracy [46]. All free induction decays were zero-filled to 250 K data points. The singlet produced by the 4,4-dimethyl-4-silapentane-1-sulfonic acid (DSS) methyl groups was used as an internal standard for chemical shift referencing (set to 0 ppm). The ^1^H NMR spectra were processed and analyzed using a Bayesil automated analysis software package, which allows for qualitative and quantitative analysis of an NMR spectrum by automatically and semi-automatically fitting spectral signatures from an internal database to the spectrum using a custom metabolite library [47]. Each spectrum was further inspected by an NMR spectroscopist to minimize compound misidentification and misquantification.

### 3.4. Data and Statistical Analysis

The metabolite data were imported into MetaboAnalyst 4.0 (https://www.metaboanalyst.ca) [48] for multivariate analysis. Data were first log-transformed and pareto-scaled [49]. Partial least squares discriminant analysis (PLS-DA), a tool used to optimize separation between different groups of samples [50], was used to visualize the differences between the control group and each of T, CL, and CL + SCFP groups. A permutation test (*n* = 2000) was performed to establish whether the observed discriminations between the groups were valid [47].

The metabolite data were subjected to statistical analysis using the GLIMMIX procedure of SAS version 9.4 (SAS Institute Inc., Cary, NC, USA). The model used for the analysis included the fixed effects of treatment, experimental period, interaction of treatment and experimental period, random effects of cow, square, interaction of treatment and square, and interaction of experimental period and square, respectively. Denominator degrees of freedom were estimated by the Kenward-Roger option in the MODEL statement. Preplanned nonorthogonal contrasts that were examined to compare means included the following: control vs. T, control vs. CL, and control vs. CL + SCFP. Significance was declared at *p* ≤ 0.05, and tendencies to significance were declared at 0.05 < *p* ≤ 0.10.

Biomarker analysis based on receiver operating characteristic (ROC) curves was performed for control and T groups using MetaboAnalyst 4.0 to identify biomarker candidates of aflatoxin ingestion in dairy cows fed no sequestering agents. Area under the curve (AUC) from ROC curve, a numerical value that indicates the relationship between sensitivity and specificity for a given diagnostic test [51], and fold change (FC) analysis (mean concentration of metabolite in T group/mean concentration of metabolite in C group) were the metrics used to interpret the performance of different biomarkers. An AUC close to 1 indicates an effective sensitivity and specificity [51]. Therefore, the most important biomarker candidate was selected on the basis of FC > 1.2 and AUC greater than 0.8 [52].

## Figures and Tables

**Table 1:** The concentrations (µM) of plasma metabolites affected by dietary treatment in dairy cows challenged with aflatoxin B_1_ with or without clay and SCFP ^1^-based sequestering agents.

Item	Treatment ^2^				SEM	Control vs. T	Control vs. CL	Control vs. CL + SCFP
	Control	T	CL	CL + SCFP				
l-Glutamic acid	295	271	274	374	17.2	0.35	0.41	0.01
l-Alanine	395	310	370	590	23.1	0.01	0.38	0.01
l-Leucine	297	229	297	387	14.7	0.01	0.99	0.01
l-Valine	382	347	375	468	19.6	0.06	0.78	0.01
l-Proline	153	133	154	205	9.67	0.15	0.91	0.01
l-Threonine	143	126	133	188	10.2	0.27	0.51	0.01
l-Isoleucine	135	131	137	172	9.09	0.71	0.92	0.01
l-Serine	148	160	147	179	10.5	0.36	0.94	0.03
l-Arginine	128	96	131	159	12.7	0.07	0.86	0.09
l-Phenylalanine	72.1	71.1	68.4	85.5	4.46	0.89	0.57	0.05
d-mannose	68.8	66.9	66.6	78.8	2.87	0.65	0.61	0.02
l-Aspartic acid	23.5	26.9	29.5	43.5	4.78	0.62	0.39	0.01
Acetic acid	1348	973	1258	1340	103	0.02	0.54	0.95
Sarcosine	1.83	1.74	1.50	1.95	0.12	0.59	0.07	0.45
Ethanol	6.81	19.9	6.38	5.19	9.29	0.01	0.85	0.26
l-Lysine	165	180	171	208	14.4	0.50	0.81	0.05

^1^*Saccharomyces cerevisiae* fermentation product–based sequestering agent (Diamond V, Cedar Rapids, IA, USA). ^2^ T = control diet + AFB_1_ (1725 μg/d); CL = T + 200 g/d of sodium bentonite clay; CL + SCFP = CL + 35 g/d of *Saccharomyces cerevisiae* fermentation product.

**Table 2:** Identification of candidate biomarkers of aflatoxin ingestion in dairy cows fed no sequestering agents.

Item	AUC ^1^	*p*-Value ^2^	FC ^3^	Sensitivity	Specificity
Acetic acid	0.91	0.01	0.72	1.0	0.8
l-Arginine	0.89	0.04	0.75	0.9	0.9
Ethanol	0.88	0.02	3.56	0.9	0.9
l-Alanine	0.86	0.01	0.78	0.9	0.9
l-Methylhistidine	0.86	0.01	0.88	0.8	0.8
l-Proline	0.81	0.04	0.89	0.8	0.8

^1^ Area under the curve from receiver operating characteristic. ^2^ Treatment effect result from *t*-test of control vs. toxin treatment. ^3^ Fold change (ratio of average metabolite concentration in cows fed toxin to average metabolite concentration in cows fed control diet).

## Supplementary Materials

The following are available online at http://www.mdpi.com/2072-6651/10/12/545/s1, Table S1: The concentration of all identified metabolites (±SD), Figure S1: Loading plots of the partial least squares discriminant analysis (PLS-DA) model for Control and Toxin (T) groups (**A**), Control and Clay (CL) groups (**B**), and Control and CL + *Saccharomyces cerevisiae* fermentation product (SCFP) groups (**C**).
