# Supplementary material for: Biomarker of Aflatoxin Ingestion: 1H NMR-Based Plasma Metabolomics of Dairy Cows Fed Aflatoxin B1 with or without Sequestering Agents
Source: Toxins (Basel). 2018 Dec 18;10(12):545. doi: 10.3390/toxins10120545 (PMC6316819; doi:10.3390/toxins10120545)
Supplement: Supplementary file 1 [file toxins-10-00545-s001.zip › toxins-399303-supplementary materials/toxins-399303-Figure S1.docx]

Supplementary Materials: Biomarker of Aflatoxin Ingestion: ^1^H NMR-Based Plasma Metabolomics of Dairy Cows Fed Aflatoxin B_1_ with or without Sequestering Agents

Ibukun Ogunade, Yun Jiang, James Adeyemi, Andre Oliveira, Diwakar Vyas and Adegbola Adesogan

| A | 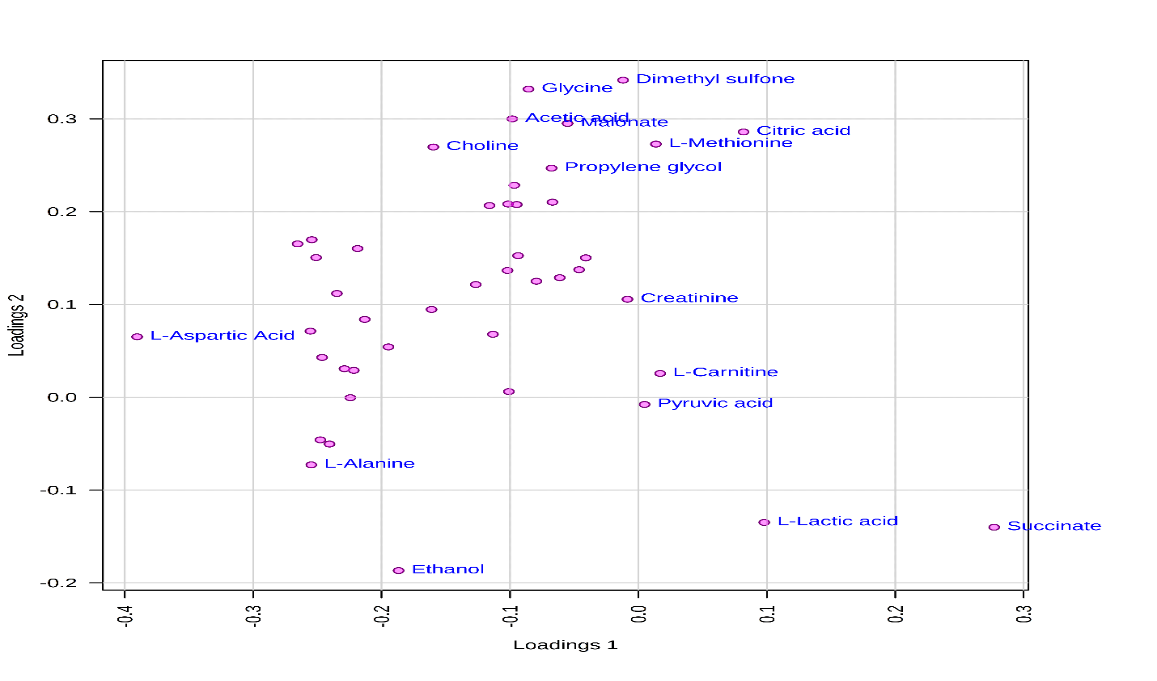 |
| --- | --- |
| B | 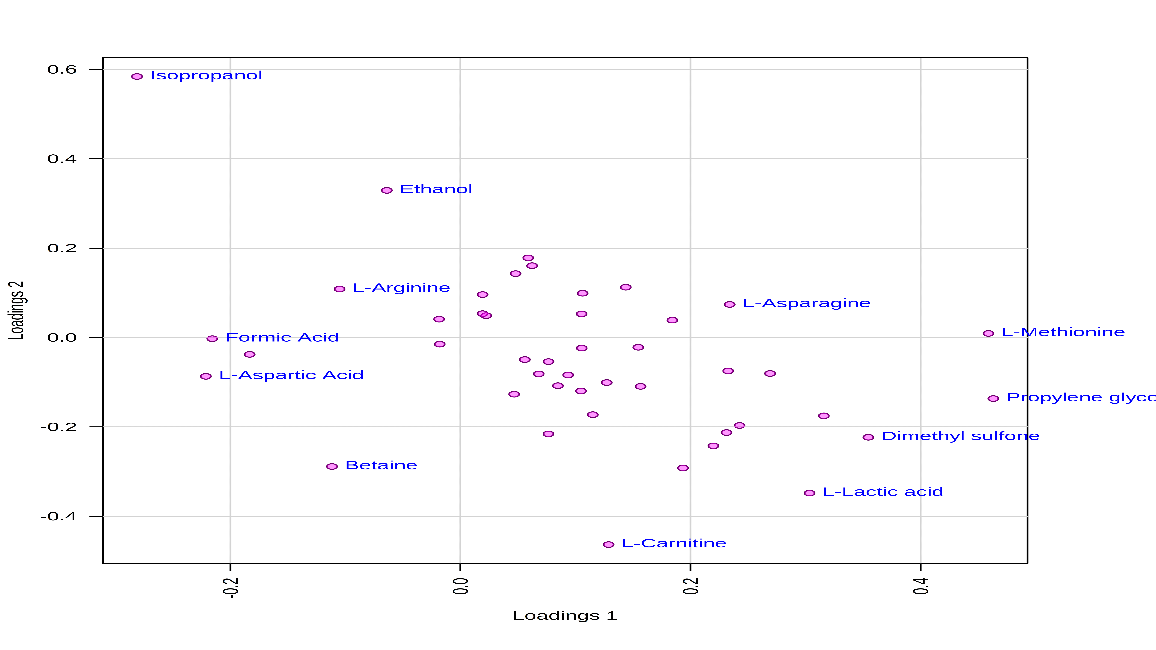 |
| C | 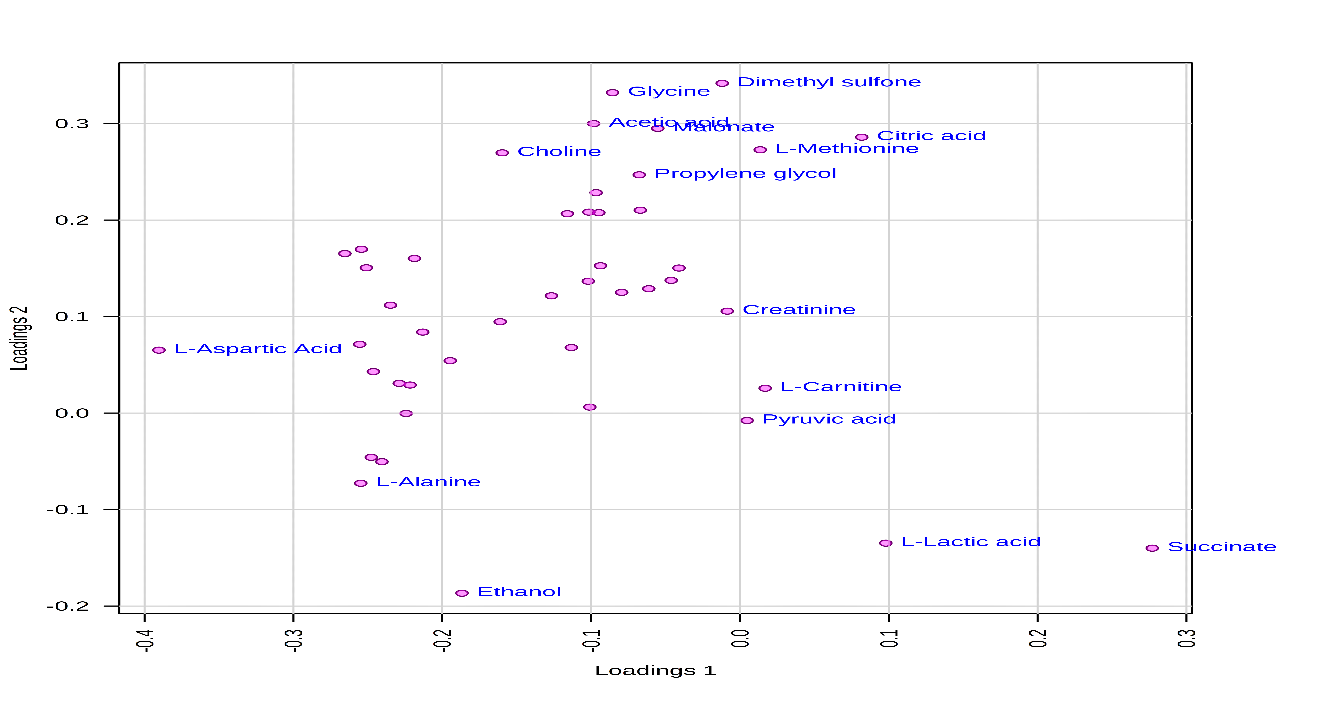 |

**Figure S1.** Loading plots of the partial least squares discriminant analysis (PLS-DA) model for Control and Toxin (T) groups (**A**), Control and Clay (CL) groups (**B**), and Control and CL + *Saccharomyces cerevisiae* fermentation product (SCFP) groups (**C**).
